# Can Professionals Resist Cognitive Bias Elicited by the Visual System? Reversed Semantic Prime Effect and Decision Making in the Workplace: Reaction Times and Accuracy

**DOI:** 10.3390/s24123999

**Published:** 2024-06-20

**Authors:** Carlotta Acconito, Laura Angioletti, Michela Balconi

**Affiliations:** 1 International Research Center for Cognitive Applied Neuroscience (IrcCAN), Università Cattolica del Sacro Cuore, Largo Gemelli 1, 20123 Milan, Italy; laura.angioletti1@unicatt.it (L.A.); michela.balconi@unicatt.it (M.B.); 2 Research Unit in Affective and Social Neuroscience, Department of Psychology, Università Cattolica del Sacro Cuore, Largo Gemelli 1, 20123 Milan, Italy

**Keywords:** semantic priming task, decision making, organisations, professionals, workplace

## Abstract

Information that comes from the environment reaches the brain-and-body system via sensory inputs that can operate outside of conscious awareness and influence decision processes in different ways. Specifically, decision-making processes can be influenced by various forms of implicit bias derived from individual-related factors (e.g., individual differences in decision-making style) and/or stimulus-related information, such as visual input. However, the relationship between these subjective and objective factors of decision making has not been investigated previously in professionals with varying seniority. This study explored the relationship between decision-making style and cognitive bias resistance in professionals compared with a group of newcomers in organisations. A visual “picture–picture” semantic priming task was proposed to the participants. The task was based on primes and probes’ category membership (animals vs. objects), and after an animal prime stimulus presentation, the probe can be either five objects (incongruent condition) or five objects and an animal (congruent condition). Behavioural (i.e., accuracy—ACC, and reaction times—RTs) and self-report data (through the General Decision-Making Scale administration) were collected. RTs represent an indirect measure of the workload and cognitive effort required by the task, as they represent the time it takes the nervous system to receive and integrate incoming sensory information, inducing the body to react. For both groups, the same level of ACC in both conditions and higher RTs in the incongruent condition were found. Interestingly, for the group of professionals, the GDMS-dependent decision-making style negatively correlates with ACC and positively correlates with RTs in the congruent condition. These findings suggest that, under the incongruent decision condition, the resistance to cognitive bias requires the same level of cognitive effort, regardless of seniority. However, with advancing seniority, in the group of professionals, it has been demonstrated that a dependent decision-making style is associated with lower resistance to cognitive bias, especially in conditions that require simpler decisions. Whether this result depends on age or work experience needs to be disentangled from future studies.

## 1. Introduction

Information that comes from the environment reaches our brain-and-body system via sensory inputs that can operate outside of conscious awareness and influence decision processes in different ways. Indeed, our perception can be influenced by environmental information on a conscious and unconscious level in all contexts and situations of daily life, including experiences concerning the workplace, and can be subject to various forms of bias.

Several lines of research have previously studied the impact of biases on human cognition [1,2,3], identifying them as systematic errors of judgment and deviations from rationality that are always present in human decision making [4]. This is especially relevant in organisations, where professionals are frequently required to make decisions in complex situations, often with limited knowledge and under time pressure [5]. But are the decision-makers in various professional positions and levels within companies conscious of the subjective elements that intervene in the decision, and are they able to counteract implicit bias?

According to ì [6], there are five situations in which decisions can be influenced by implicit and unconscious processes. The first scenario involves an event or criterion in the environment that is not consciously perceived by the decision-maker, while the second one concerns a lack of awareness regarding the contingencies or relationships between the cue and the outcome. Lack of awareness of the cues relied on when making a judgement or decision, or the use of the cues themselves are other third and fourth manners in which unconscious influence can occur. The fifth circumstance is a lack of awareness of choosing or deciding.

A decision, therefore, can be influenced at different levels of processing, such as during perceptual stages or semantic representations [7]. An influence at the perceptual level can lead to perceptual bias, which occurs when an individual’s internal state alters the perception of an external stimulus (in terms of colour or size or motion or symmetry). Perceptual bias can also occur in response to more complex stimuli, such as human faces and everyday objects [8]. On the other hand, an influence during semantic representations of a stimulus leads to semantic bias, which involves extracting meaning from the stimulus itself [9].

Regardless of the level at which sensory environment information influences decision-making behaviour, this process is affected by two types of variables: context-individual-related factors (i.e., observer’s attention, expectation) [10] and stimulus-related information (i.e., features of the stimulus) [11]. 

Firstly, regarding context-individual-related factors, the interpretation of the same external stimulus may be interpreted differently according to the individual’s characteristics [12,13]. For instance, our choice behaviour can also be influenced by individual differences in our decision-making style [14,15], which are usually investigated through self-report questionnaires, such as the General Decision-Making Scale (GDMS) [16,17], which allows profiling decision-makers according to different decision styles. 

Secondly, concerning the stimulus-related information, the stimulus properties can influence voluntary and involuntary attentional processes, thereby influencing the salience and relevance of stimuli and, accordingly, the decision [18]. Also, a crucial variable related to the stimulus is anticipation, which influences perception based on the likelihood of encountering specific stimuli [18]. 

Concerning anticipation, there is a prototypical effect, that is the “priming effect”, in which an individual’s exposure to a certain stimulus (defined as prime) influences their response to a subsequent stimulus (defined as probe or target), without any awareness of the connection between the two stimuli. Consequently, in this condition, decision making is no longer an explicit, rational, and conscious process; rather, it is guided and influenced implicitly and unconsciously by exposure to the prime stimulus, which operates subliminally. 

Several studies have examined how this process of influence occurs [19,20]. For instance, the “situated interference model of priming” [20] posits that, regardless of whether the prime stimulus is processed consciously or unconsciously, there are three main stages that must be taken into account when considering the priming effect and prime stimulus’ function concerning decision making: (i) the “prime exposure”, in which the prime stimulus activates related information in memory; (ii) the “misattribution” stage, in which this information is incorrectly attributed to a person’s natural response to an object or situation; and (iii) the “afforded questions”, in which the misattributed content is used to make a decision. During the first stage, the prime makes information about the construct itself accessible to memory at the experiential, semantic, or evaluative level. However, this change in informational accessibility does not directly influence behaviour. During the second stage, the information made accessible by the prime is attributed to its internal thought process, becoming a source of bias in decision-making processes. In the third step, the misattributed information is used to respond to different questions posed by the current situation, altering the information considered during the decision-making process. 

An example of a priming effect in which the incorrect attribution of the information is recalled by the prime is named “semantic priming”, which normally occurs when a word or picture (probe) is preceded by a semantically related stimulus (prime) [21]. The semantic priming effect refers to the faster and more accurate response to a target word when it is preceded by an associatively/semantically related word than when it is preceded by an unrelated word.

When semantic priming elicits an implicit response that interferes with the correct processing of information, it represents a cognitive control challenge known as stimulus conflict. Stimulus conflict occurs when the presence of task-irrelevant perceptual information in the prime stimulus compromises with the processing of task-relevant information. Therefore, coping with this situation may require the activation of explicit control processes to reduce the negative impact of implicit processing mechanisms on performance [22].

Moreover, this ability is particularly important when the characteristics of the environment present barriers to goal achievement [22], as in organisational settings. Indeed, the organisational environment is a concrete example of how diverse stimuli can influence decision-making processes by inducing cognitive biases. Moreover, in this context, it is necessary to cope with situations of constant change within and without the organisation, effectively implement new strategies, and operate under uncertain conditions [12]. 

The ability to contrast the semantic priming effect is a cognitive activity dependent on a variety of cognitive and neural processes, including sensory processing and data processing. In this context, age could be one of the aspects that influence performance in decision making that must be considered [23]. Several studies indicate that, with getting older, many cognitive abilities, including processing speed and visual perception, deteriorate gradually, and that this impacts cognitive tasks performance [24,25,26,27]. However, most of the evidence on the impact of ageing in semantic priming concerns lexical decision tasks [27,28]. Also, a recent study showed a lack of significant differences between youths and adults in priming effect [29]. 

Based on this theoretical framework, the current study aimed to investigate the decision-making processes when influenced by external stimuli that affect subthreshold unconsciously and implicitly and the ability to resist biases derived from a “picture–picture” semantic priming task by comparing a group of professionals with a group of newcomers in organisations, namely, young individuals entering the labour market.

The novel semantic priming task designed for this study was characterised by two different conditions (i.e., a congruent and an incongruent condition): in both conditions, the participant was asked to detect whether there was an image of an animal in the probe stimulus.

The two different conditions were based on the presence and absence of semantic relationships between the prime and probe. Specifically, in the congruent condition, the prime stimulus is represented by an image of an animal and the probe stimulus consists of the simultaneous appearance of six images, including one animal and five objects. In this condition, therefore, there is semantic congruence between the prime and the probe, both belonging to the same semantic group (animal–animal), and this can facilitate cognitive processing and reduce reaction times (RTs) to the task. Instead, in the incongruent condition, the prime stimulus is represented by an image of an animal and the probe stimulus consists of the simultaneous appearance of six images, all depicting objects. In this condition, therefore, there is semantic incongruence between the prime and the probe, belonging to two different semantic groups (namely, animal and object), and this requires the inhibition of implicit processing of the prime.

For each trial, two types of behavioural data were collected. The first measure concerns the participant’s behavioural accuracy (ACC). In addition, the RTs to each trial were also collected, as the time taken by the participant to respond is an indirect measure of the workload and cognitive effort required by the task [30,31,32,33,34].

Moreover, to integrate the behavioural measures, the GDMS questionnaire was administered to assess individuals’ differences in their decision-making styles [16,17]. The GDMS was adopted to explore potential associations between five different decision-making styles (rational, intuitive, avoidant, dependent, and spontaneous) and the ability to resist cognitive bias. 

Regarding behavioural data, it was hypothesised that the incongruent condition may require greater RTs compared to the congruent condition, due to the need to inhibit the automatic response elicited by the prime to respond correctly to the task. Since the implicit and unconscious processing of the prime involves activation of the semantic nodes associated with it, in the incongruence condition, it is necessary to inhibit the response facilitated by the animal prime and employ an explicit and conscious processing of the probe stimulus, which requires greater cognitive effort and RT.

Considering the need to inhibit the implicit response supported by the prime stimulus, it is also plausible to presume that the incongruent condition may result in a decrease in ACC. Specifically, the decision-making complexity imposed by the task, especially about the incongruent condition, could be a critical factor for newcomers, who are less experienced in making decisions in complex situations that require the integration of multiple factors. Therefore, it is expected that, compared to professionals, the newcomers could display less ACC and greater RTs during the incongruent condition, given the difficulties they could encounter in the decision-making process and their lower level of expertise and seniority. 

Concerning self-report data, we can suppose significant correlations between GDMS-dependent decision-making style and behavioural data. Indeed, the GDMS-dependent decision-making style is particularly interesting to this study because it is related to the difficulty of carrying out a decision-making process without being worried and disturbed by external thoughts [14] and is also linked to nonadaptive coping strategies [35]. Therefore, it could be supposed that people who report lower ACCs and longer RTs are predominantly characterised by a dependent decision-making style, in which decision-making is influenced and interfered with by the prime, as well as by the continuous search for external reinforcement.

## 2. Materials and Methods

### 2.1. Sample

Sixty-one healthy participants (male = 21; female = 40, M_age_ = 34.58, SD_age_ = 11.44); 29 professionals (male = 17; female = 12, M_age_ = 38.98, SD_age_ = 10.87); and 32 young people approaching the world of work, here considered as “newcomers” (male = 9; female = 23, M_age_ = 34.21, SD_age_ = 11.32) were recruited and provided written informed consent for this study. Before starting the participants’ recruitment, a priori power analysis was conducted to determine the minimum sample size needed to test the research hypotheses. The analysis performed with the G*Power 3.1.9.7 software [36,37] indicated that a sample size of 40 individuals could be enough for repeated-measures ANOVA with two measurements and two groups (effect size f = 0.40, α = 0.05, power = 0.80), in order to obtain within- or between-subjects effects. Thus, the sample size of N = 61 was considered adequate to test the research hypotheses.

Specifically, the group of professionals was composed of individuals with a minimum expertise of 3 years, while the newcomers where in the organisations for less than 1 year. The group of professionals was engaged in collaboration with different medium-to-large Italian companies from different sectors. Additionally, to increase the generalisability of the results and avoid possible bias, the professionals had various professional specialisations, as they worked in different job roles, such as infrastructure management, engineering management, human resource management, training, and professional development. The second group consisted of individuals at the end of their studies, who had graduated within the past two years and who were entering the labour market as newcomers in the organisations. All participants had normal to correct vision, did not suffer from severe levels of depression, and did not have a history of neurological or psychiatric disorders. The presence of low cognitive functioning, as well as being in therapy with psychoactive drugs that could alter cognitive or decision-making abilities, but also abnormalities in short- or long-term memory, represent the exclusion criteria from the study. Therefore, the participants’ cognitive level was medium to high, and they had no executive function impairments that could impact their ability to perform the tasks. In addition, both groups had a minimum schooling level of eighteen years, as all participants had master’s degrees. Some of the professionals also had higher qualifications, such as a doctoral title. Additionally, no participants reported a level of familiarity with the task, which was created ad hoc for this study. The study was carried out in accordance with the Declaration of Helsinki (2013), according to the GDPR—Reg. UE 2016/679 and its ethical guidelines, and approved by the Ethics Committee of the Catholic University of the Sacred Heart, Milan, Italy.

### 2.2. Procedure

**Figure 1:**
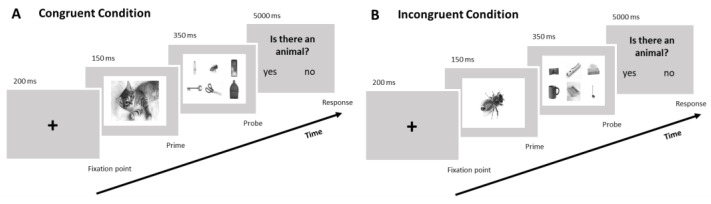
Semantic priming decision-making task. Examples of congruent (**A**) and incongruent (**B**) conditions of the experimental task. After the instructions screen, each trial began with a fixation point (200 ms), and then an animal prime stimulus was shown for 150 ms. Participants then viewed a screen with six different images for 350 ms and had up to 5000 ms to press one of the two keys on a keyboard to indicate if one of the six pictures represented an animal.

The experiment procedure took approximately 10 min and was conducted in a quiet room, where participants were instructed to sit on a comfortable chair approximately 80 cm away from a computer monitor. A novel semantic decision-making task with two different conditions (congruent and incongruent conditions) was created, and it was administered via a web-based experiment-management platform (PsyToolkit, version 3.4.4) [38,39]. All participants performed 16 trials in each of the two conditions: trials within each session were presented randomly (see Figure 1 for task description). Participants were required to select the key corresponding to the letter L if an animal was present and the key corresponding to the letter K if there was no animal among the six images displayed simultaneously. If any of the six images depicted an animal, the trial was classified as satisfying the congruent condition. In contrast, if the six images depicted only objects, the trial would be considered an incongruent condition.

The novelty of this task consists of the semantic incongruence condition, which may be utilised as a helpful and unique technique to test resistance to cognitive bias. Indeed, the semantic incongruence between the prime (animal) and the probe (objects) stimulus requires the ability to inhibit the response facilitated by the prime together with an explicit and conscious processing of the probe stimulus to provide the correct response to the task.

Behavioural performance data for all subjects in terms of ACC and RTs was collected for each of the two conditions. Specifically, ACC was determined as the ratio of the number of correct responses to the number of possible responses. The RTs correspond to the milliseconds used in each trial by the participant, in a time window of a maximum of 5000 ms, to answer the question “Is there an animal?” by pressing the key on the keyboard. For purposes of analysis, only correct responses RTs were considered.

### 2.3. Self-Report Scale

The Italian version of the GDMS [16,17] was administered to measure individuals’ decision-making styles. This scale is composed of 25 items, divided into 5 sub-scales, for which participants are asked to indicate their level of agreement on a five-point Likert scale. The five sub-scales correspond to the five different profiles of decision-making style: (i) rational, characterised by a deep search for information, considering all alternatives and their repercussions; (ii) intuitive, typical of an individual who focuses on global aspects and tends to make decisions based on intuition; (iii) dependent, that prefer to receive suggestions and advice to make a decision; (iv) avoidant, characterised by a tendency to avoid making decisions; (v) spontaneous, typical of those feeling of immediacy and a desire to come through the decision-making process as quickly as possible.

### 2.4. Data Analysis

Concerning the analysis of behavioural data, two repeated measures analysis of variance (ANOVAs) were applied to ACC and RT indices. 

Specifically, ANOVA with *Group* (2: professionals, newcomers) as the between-subject factor and with *Condition* (2: congruent, incongruent) as the independent within-subject factor was performed for behavioural data (ACC and RTs).

In case of significant effects, pairwise comparisons were applied to the data. Simple effects for significant interactions were further checked via pairwise comparisons, and Bonferroni correction was used to reduce multiple comparisons potential biases. For all the ANOVA tests, the degrees of freedom were corrected using Greenhouse–Geisser epsilon where appropriate. Furthermore, the normality of the data distribution was preliminarily assessed by checking kurtosis and asymmetry indices. The size of statistically significant effects was estimated by computing partial eta squared (η_p_^2^) indices.

Before applying the correlations, as a preliminary analysis, we verified that there were no significant differences in the scores of the GDMS subscales between the two groups. Pearson correlations, with Bonferroni corrections for multiple comparisons, were applied to GDMS subscale scores and behavioural data (ACC and RTs) for each condition (congruent and incongruent), considering the two groups of participants.

## 3. Results

### 3.1. Behavioural Data

**Figure 2:**
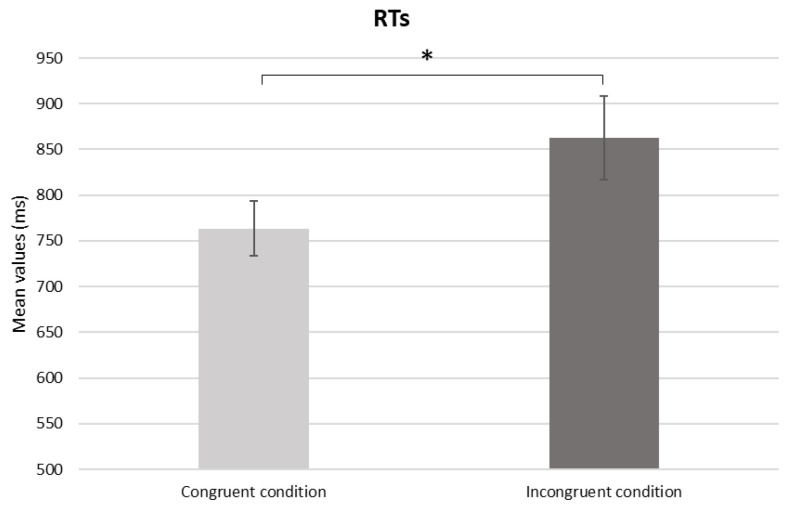
Behavioural results. Bars show significant differences for RTs in the congruent condition compared to the incongruent one. Bars represent ±1 SE. Star marks statistically significant pairwise comparisons.

Regarding the ANOVAs performed on behavioural data, a significant main effect in the within-subject factor *Condition* was found (*F*(1,56) = 4404, *p* = 0.040, *η_p_^2^* = 0.073) for RTs, with an increase in it in incongruent conditions compared to the congruent one (*p* = 0.040) (Figure 2).

No other significant differences were found. ANOVA performed on ACC was not significant.

### 3.2. Correlation Results

**Figure 3:**
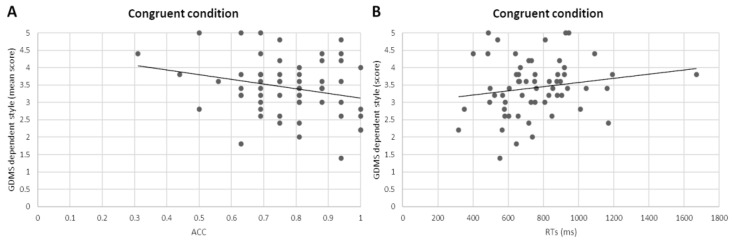
Pearson correlations. The scatter plots display (**A**) a significant negative correlation between dependent decision-making style and ACC in the congruent conditions and (**B**) a significant positive correlation between dependent decision-making style and RTs in the congruent conditions.

The Pearson correlation performed between GDMS subscale scores and behavioural data (ACC and RTs) for each condition (congruent and incongruent) on the two groups of the sample showed a negative correlation between the dependent decision-making style and ACC in the congruent condition (r = −0.433, *p* = 0.021) and a positive correlation between dependent decision-making style and RTs in the congruent condition (r = 0.431, *p* = 0.022) for the professionals’ group (Figure 3).

No other significant correlations were found.

## 4. Discussion

This research examined the relation between decision-making style (GDMS subjective scores) and cognitive bias resistance during a decision-making task in a group of professionals compared with a group of newcomers. The influence of subthreshold external stimuli acting unconsciously and implicitly on decision-making processes was explored thanks to a semantic priming task, through which behavioural objective performance was evaluated by considering ACC and RT scores.

Behavioural data revealed that professionals and newcomers display the same ACC in congruent and incongruent task conditions. This result could indicate that professionals showed adequate cognitive control, especially in semantic incongruent conditions, when the activation of explicit control was needed to reduce the impact of implicit priming mechanisms to complete the decision process [22]. Also, the absence of differences between professionals and newcomers could suggest similar skills in resisting cognitive biases, perhaps not influenced by age and professional background [29]. Otherwise, there might be a ceiling effect due to the task’s simplicity: to confirm this interpretation, future studies could apply the same research protocol in distinct professional contexts with a larger sample size.

Even though the different semantic conditions between the prime and probe do not impact ACC, the significant differences in RTs observed for the incongruent compared to the congruent condition suggest a greater effort to provide an accurate response in the incongruent condition. Besides the notion that RTs are indices of cognitive effort and mental load, it is possible to suppose that participants in the semantic incongruent condition were able to implicitly detect the incongruency at the “misattribution” stage of the priming process, as defined by the “situated interference model of priming” [20], that is, the stage in which the information enabled by the prime could incorrectly facilitate the response. The increase in RTs could therefore reflect the cognitive processing effort in inhibiting the prime response, employing conscious thinking and actively counteract the cognitive bias induced by viewing the prime stimulus (i.e., incongruent condition). The novelty of this task consists of this peculiar semantic incongruent condition, which proved to be useful in testing the resistance to cognitive bias, although not differentiating between the two groups.

In addition to previous evidence suggesting that there are no significant differences between youth and adults in priming effect [29], this finding demonstrates that there would appear to be no differences in terms of seniority as well. Indeed, it is worth noticing that RTs increased for both professionals and newcomers, a result that can be interpreted together with the accuracy finding. Resistance to cognitive bias does not depend on the individual’s seniority but rather may depend on the stimulus property: dealing with implicit cognitive bias and making an accurate decision under conditions of incongruence is more time-consuming for professionals at all levels.

In addition, the correlations performed between the self-report measures of decision style and the behavioural data derived from the task were useful to explore in terms of the influence of context-individual-related factors in the two groups. A previous study suggested that individual differences in decision-making styles can influence decision-making behaviour [10]; however, the relation between individual decision style and cognitive bias under decision-making conditions had not been explored in professional contexts before.

Interestingly, correlations showed that decision styles are more influential among professionals than newcomers, especially in semantic congruent conditions. Indeed, for the group of professionals, we observed a negative correlation between the GDMS-dependent decision-making style subscale and ACC in the semantic congruent condition. A possible way of interpreting this result could be that high scores of dependent decision-making style are associated with lower ACC in the performance of semantic congruent trials. In addition, the more the professionals score high in the dependent decision-making style, the longer time they took to provide the correct answer in the semantic congruent trials, as shown by a positive correlation between dependent decision-making style and RTs in the semantic congruent condition for the professionals’ group.

Despite the lack of studies linking decision-making style with cognitive bias derived from a semantic priming task, the behavioural characteristics typically associated with the dependent style may provide some insights for understanding current findings. It was demonstrated that dependent decision-making style may be associated with the difficulty of making decisions without being influenced by distracting thoughts [14]. In addition, it has been shown that this style of decision-making is also characterised by low self-esteem, which is linked to nonadaptive coping strategies [35] that may impair the ability to act decisively. Thus, a possible interpretation of results could be that having a dependent decision-making style may be associated with a lower ability to exert an individual top-down executive control that turns into a lower behavioural performance, even in simple (i.e., congruent) conditions.

On the other hand, it might be plausible that professionals navigating dynamic and fast-paced organisational environments develop the tendency to take decisions always considering the external context and others’ advice, as if the work environment itself may be responsible for the increased use of a dependent decision-making style, reflected in the need to take more time before responding to any decision-requiring task. The other side of the coin may be that this decision-making style can also result in a decreased capacity to act decisively and become a victim of a form of reinforcing bias. To disentangle the role of the decision styles and the actual behaviour of professionals, further studies are needed to deepen the relationship between decision-making styles, executive functions, and the work environment.

Taken together, these findings suggest that, under the incongruent decision condition, the resistance to cognitive bias requires the same level of cognitive effort, regardless of seniority. However, with advancing seniority, in the group of professionals, it has been demonstrated that a dependent decision-making style is associated with lower resistance to cognitive bias, especially in conditions that require simpler congruent decisions. Whether this result depends on age or work experience needs to be disengaged from future studies.

At the methodological level, the current results outlined how this novel semantic priming task can be a useful tool for studying resistance to cognitive bias and professionals’ ability to inhibit implicit and unconscious decision-making processes with the advantage of conscious and detailed processing. Indeed, cognitive control, which can be defined as the ability to modulate or manipulate explicit, bottom-up neural processes in pursuit of goals, appears as a central and indispensable aspect of high-level cognition for decision making in complex environments and could be a target function to be explored in relation to decision making in organisational settings.

Some limitations should be considered for future studies. Firstly, the sample size can be increased and balanced for gender to improve the representativeness and reliability of the results and disclose potential gender differences in the ability to resist cognitive bias. Secondly, to deeply explore the implicit components of a decision process, it could be useful to adopt a neuroscientific approach [40,41,42] that integrates behavioural data with neuroscientific measures such as electroencephalogram [43] and autonomic measures recording [44] to map the neuro- and psycho-physiological response during the decision-making process, highlighting levels of cognitive effort and emotional engagement [45,46,47,48,49,50]. Indeed, the integration of neuro- and psycho-physiological measurements with behavioural and self-report data represents a useful approach to prevent bias, such as social desirability biases [51,52]. Additionally, to control if the ability to resist bias is related to the family of stimuli, it might be useful to add an active control condition with a prime stimulus represented by an object, or even consider perceptually different or more ecological stimuli, belonging to the organisation context. Finally, to investigate whether and how the relationship between decision-making styles and the ability to resist cognitive bias may change in a subject as their professionality increases, future research could conduct longitudinal studies.

## Data Availability

The data presented in this study are available on request from the corresponding author due to ethical reasons for sensitive personal data protection (requests will be evaluated according to the GDPR—Reg. UE 2016/679 and its ethical guidelines).
